# Diagnosis of Diamond-Blackfan anemia in adulthood: case series and review of the literature

**DOI:** 10.1186/s13023-024-03490-6

**Published:** 2024-12-19

**Authors:** Francesco Versino, Paola Bianchi, Elisa Fermo, Wilma Barcellini, Bruno Fattizzo

**Affiliations:** 1 Hematology Unit, Fondazione IRCCS Ca’ Granda Ospedale Maggiore Policlinico, Milan, Italy; 2 Department of Oncology and Hemato-oncology, University of Milan, Milan, Italy; 3 Fondazione IRCCS Ca’ Granda Ospedale Maggiore Policlinico, via Francesco Sforza 35, Milan, 20100 Italy

**Keywords:** DBA, Anemia, Inherited bone marrow failure

## Abstract

Diamond–Blackfan anemia (DBA) is a rare constitutional inherited bone marrow failure syndrome (iBMF) characterized by progressive severe non-regenerative anemia and congenital abnormalities. Diagnosis is made by identification of a DBA-causing variant, typically in a ribosomal protein gene. More than 99% of patients are diagnosed in the pediatric age, but clinical manifestation may be mild and severe anemia can occur later in the patient’s life. Moreover, the expanding availability of molecular testing is increasing the ability to identify DBA variants also in adults with a non-canonical DBA phenotype. Therefore, adult hematologists must maintain a high clinical suspicion and awareness towards possible DBA diagnosis in adulthood. In this context, the most common differential diagnoses are acquired BMFs such as pure red cell aplasia (PRCA) or hypoplastic myelodysplastic syndrome (MDS). Here, we present three adult patients diagnosed with DBA, where the identification of the causative mutation occurred several years from PRCA misdiagnosis or was made after screening for an affected relative. We also provide a review of 16 cases available in the literature and give hints on possible treatment strategies.

## Introduction

Diamond–Blackfan anemia (DBA) is a rare constitutional inherited bone marrow failure syndrome characterized by progressive severe non-regenerative anemia with erythroblastopenia and tendency to develop secondary malignancies such as colorectal cancer, osteosarcoma or myeloid neoplasia [1–4]. About 80% of cases of DBA are related to a heterozygote mutation in ribosomal protein (RP) genes of either the small or large protein subunit, inherited in an autosomal dominant fashion. As of now, more than 20 RP genes have been identified, the most common being *RPS19*, *RPL5* and *RPL11*; moreover, also X linked mutations on non-RP genes such as *GATA1* or *TSR2* have been described as associated with DBA. Clinical manifestations can be extremely variable, also due to the incomplete penetrance of the mutation and to the lack of a clear genotype-phenotype correlation. Typically, the diagnosis is made at 2–3 months of life and 99% of patients are diagnosed before the age of 5 [1]. In children, the mainstay of treatment are steroids and supportive therapy with transfusion and iron chelation. Hematopoietic stem cell transplant from a non-affected sibling donor (genetic study needed) or matched unrelated donor is advised especially in children less than 10 years old [1–3].

DBA diagnosis in adulthood is extremely rare and generally occurs in patients developing symptomatic hypo-regenerative anemia or in mildy symptomatic subjects, due to later identification of a DBA-causing variant during screening of an affected relative. The most common differential diagnoses (often misdiagnosis) include acquired pure red cell aplasia (PRCA) and hypocellular myelodysplastic neoplasms (MDS). In the last decade, the widespread availability of genetic testing, including targeted-next generation sequencing (t-NGS) panels and whole exome sequencing (WES), are increasing the ability to identify DBA variants in adult-patients with a non-canonical phenotype [4]. Here, we report three adult patients followed for DBA at our Center, where the identification of the causative mutation either followed or preceded by several years the onset of symptoms. A review of the most recent literature is also provided.

## Methods

Three cases of adult-onset DBA, diagnosed at a tertiary Center in Milan, Italy, from January 2023 to March 2024 were included in the study. Clinical and hematological features, including laboratory values and bone marrow characteristics, and specific treatment were retrospectively collected. Patients gave informed consent, and the study was conducted according to the Helsinki Declaration.

Clinical exome was evaluated by SureSelect Custom Constitutional Panel 17 Mb (CCP17) or CD Clinical Focused Exome – (CDCFE) (Agilent Technologies), and sequenced on Illumina NextSeq 550/2000 platforms (Illumina, San Diego, USA). Variants were called and annotated using the Alissa Align&Call and Interpret software (Agilent Technologies).

**Fig. 1:**
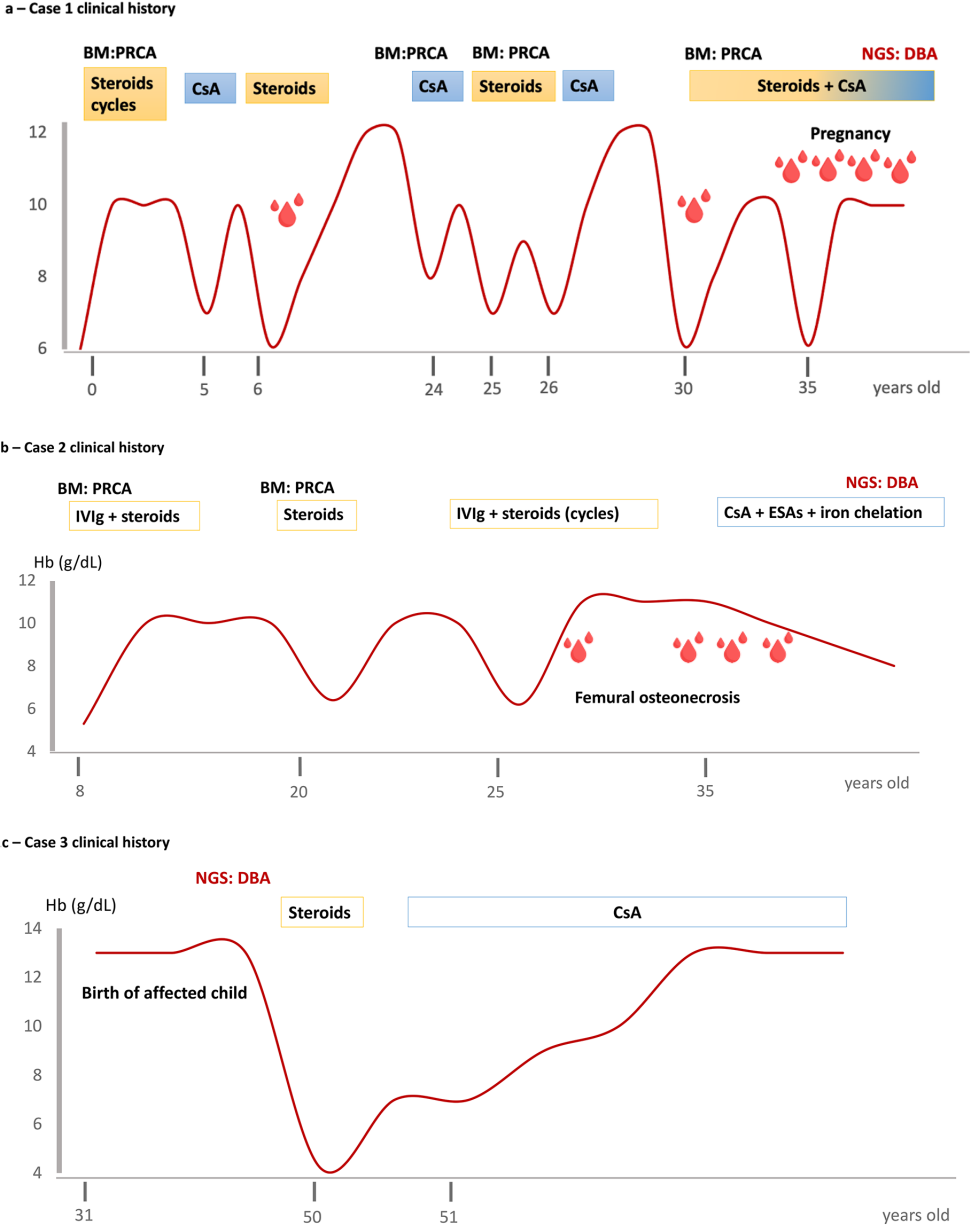
(**a**,** b**,** c**) - Case 1, 2, 3 clinical history. PRCA: Pure red cell aplasia, DBA: Diamond-Blackfan anemia, CsA: Cyclosporine A, BM: Bone marrow, IVIg: intravenous immunoglobulin, ESAs: erythropoietin stimulating agents, NGS: Next generations sequencing. Red drops represent red blood cell unit transfusion requirement

## Case report

### Case 1

A 32-year-old woman presented in 2015 to the outpatient clinic for hemolytic anemia. Figure 1a depicts the patient’s clinical history. The patient was born with no congenital abnormalities, but with severe anemia for which she underwent bone marrow evaluation consistent with pure red cell aplasia. DBA mutations had not been tested. In her first 25 years of life the patient underwent multiple steroids cycles with variable efficacy and three trials with cyclosporine A, with transient responses and re-evaluation of bone marrow confirming previous diagnosis. During follow-up, she also suffered from a cerebral vein thrombosis. Several investigations performed did not unravel the causes of anemia (MCV 110 fl., negative direct anti-globulin test, with different methods including mitogen stimulated DAT, absence of PNH clone, normal RBC membrane tests and enzyme activities). A total body CT scan showed only moderate splenomegaly, no signs of thymoma. Hemoglobin electrophoresis documented a persistence of HbF (5.5%). Bone marrow evaluation showed mildly reduced cellularity (50–60%), with normal myeloid and megakaryocytic lineages and reduced erythroid precursors, a 15% polyclonal T-cell infiltrate and presence of anti-erythroblast antibodies [5, 6]. The diagnosis of PRCA was confirmed and again treated with steroids and cyclosporine. In 2019 the patient had her first pregnancy and gave birth to a healthy child. During gestation she required multiple RBC transfusions (16 in total) and from 2022 became transfusion dependent. Evaluation of iron overload showed increased ferritin levels (> 1000 mcg/l) with mild iron deposition at liver T2* MRI, and was therefore started iron-chelation therapy. Re-evaluation at our Center showed persistent severe macrocytic anemia and bone marrow findings (including anti-erythroblast antibodies) where the same, except for an increase of the T-cell infiltrate to 25% with the appearance of a clonal T-cell large granular lymphocyte (LGL) population. Finally, exome sequencing was carried out and showed a known pathogenic variant in RPS26 gene (NM_001029.5 : c.4-1G > A) consistent with diagnosis of DBA.

### Case 2

A 36-year-old woman was referred in 2023 to our clinic due to a long history of anemia since the pediatric age, including an episode during a viral infection (Hb 5.3 g/dL) with reticulocytopenia and bone marrow evaluation consistent with diagnosis of PRCA (Fig. 1b). During this acute episode and in the following years she was treated with several cycles (10 total) of intravenous immunoglobulins +/- steroids, obtaining transient normalization of hemoglobin levels. In 2019, her anemia worsened and started to require transfusions every 6 to 8 months, coupled with immunoglobulin and steroid therapy. In 2022 she lost response to intravenous immunoglobulin, and transfusion frequency worsened, therefore therapy with recombinant erythropoietin and cyclosporine A were added, without response. Iron chelation therapy with deferasirox was started given the increased transfusion need. In 2023 she experienced avascular necrosis of the femoral head and was referred to our Center. At our evaluation she had no signs of constitutional malformations, and bone marrow evaluation showed a reduced cellularity with normal myeloid and slightly increased megakaryocytic lineages but absence of erythroid precursors and a 30% polyclonal T cell infiltrate. Whole exome sequencing showed an RPS19 mutation (NM_001321484.2: c.184 C > T, p.Arg62Trp) already reported in the literature, consistent with DBA diagnosis. Additionally, a NGS myeloid panel was negative.

### Case 3

A 50-year-old man presented in 2023 to the emergency room complaining fatigue and palpitations, the patient’s clinical history is depicted in Fig. 1c. Complete blood count (CBC) showed severe normocytic anemia, Hb 4.1 g/dL (MCV 85 fl.), with platelets and leukocytes within the normal range; hemolysis markers were also normal. He was transfused with 4 red blood cell units and discharged to the outpatient clinic. Medical history showed a diagnosis of bipolar disorder treated with sodium valproate, he had no congenital musculoskeletal abnormalities but was low in stature and had mild facial dysmorphic features. Family history revealed a 9 years-old daughter previously diagnosed in another center with RPS19-mutated (c.3G > A, p.Met1) DBA at birth and treated with transfusion and steroids achieving transfusion independence. At that time, family screening had shown that the father (our patient) was carrier for the RPS19 mutation. He showed increased adenosine deaminase activity (ADA), similarly to his daughter, but had normal Hb levels, and was not put in hematological follow up. At hematological evaluation, the patient showed severe, hyporegenerative reticulocytopenic (19 × 10^9^/L) anemia, and bone marrow evaluation showed a hypocellular bone marrow (25% cellularity) with well-represented trilineage precursors and a 15% polyclonal T-cell infiltrate. Cytogenetics was normal and NGS panel for myeloid neoplasms negative. Treatment with steroids 1 mg/kg day was attempted, although with no response at 1 month. Cyclosporine A at low dose (200 mg per day) was added obtaining near-normal Hb (12.1 g/dL) after 1 month along with transfusion independence. At last follow up after 6 months of therapy the patients has Hb 14.5 g/dL.

## Review of the literature

Table 1 presents 7 reports of 16 cases of adult DBA available in the literature. The first publication dates back to 1985, and concerns a 25-year-old man and a 64-years-old woman with long standing history of severe anemia and transfusion dependence, presenting with several musculoskeletal abnormalities (webbed neck and hand abnormalities). They were diagnosed with congenital pure red cell aplasia and responded to steroid therapy [7]. All other reports are published in the last decade and display DBA pathogenic mutations, mostly in RPL11 gene [8–13]. In the reported cases, patients present with new onset severe a-regenerative anemia in the adult age, but through accurate medical history most of them showed mild anemia since the pediatric age. Moreover, all patients had congenital malformations, the most common being thumb hypoplasia. However, diagnosis was delayed until the occurrence of severe transfusion dependent anemia without other possible causes which triggered genetic testing. Treatment strategies described are diverse and mostly rely on steroids and transfusions. One patient was treated with cyclosporine and danazol with response, one with cyclosporine single agent without response and one with eltrombopag, again without response [8, 12, 13]. Notably, a family study by Carlston et al., identified 7 adults with mild phenotype (5 with thumb hypoplasia, 1 with short stature and 1 with mild anemia and cleft palate) by studying an index 2-year-old boy with DBA [10]. Finally, a study of the German DBA registry identified 179 index DBA children and, by molecular evaluation in the parents, found 43% silent carriers of mutations with normal phenotype and no history of anemia [14].

**Table 1:** Review of the literature

Author	Year	Case report	Comments
Balaban et al. [7]	1985	64 and 25 years old patients with muscoloskeletal abnormalities and severe anemia	First report of congenital pure red cell aplasia, without genetic analysis. Response to steroid therapy.
Flores-Ballester et al. [8]	2015	35 years old with congenital thumb hypoplasia and severe anemia	De novo mutation in RLP11 gene. Transfusion independence achieved through cyclosporine and danazol treatment.
Narla et al. [9]	2016	23 years old with short stature, thumb hypoplasia and severe anemia	Intronic mutation in RPL11 gene causing a pre-rRNA maturation defect and lower mRNA expression. Clinical response to steroid therapy.
Carlston et al. [10]	2017	Family study of seven adults, 6 with congenital abnormalities and one anemic, starting from an index DBA child.	Correlation study of RPL11 gene confirms the variable penetrance of DBA. Carriers show thumb abnormalities, only one mild anemia.
Mars-Holt et al. [11]	2022	35 years old woman with spina bifida and genitourinary tract defect with severe anemia	Pathogenic variant in RPL11 gene. No response to steroid therapy, evaluation for bone marrow transplantation.
Dorenkamp et al. [12]	2023	20 years old patient with multiple congenital malformations but only mild anemia	De novo gene variant in the RPL5 gene, detected after worsening of anemia in the adult age. No response to steroids and eltrombopag.
Strasser et al. [13]	2024	55 years old patient with thumbs abnormalities and anemia	DBA mutation on RLP11 gene. No response to corticosteroids or cyclosporine and later development of pancytopenia.
Moetter et al. [14]	2011	German DBA Registry study.	The investigation of parents of 179 DBA children identified 43% silent carriers of mutations with normal phenotype and no history of anemia.

## Discussion

We presented here three cases of adult patients diagnosed with DBA at our Center. Notably, none of our patients presented the typical congenital abnormalities associated with the disease. The first patient had a longstanding history of PRCA, with presence of anti-erythroblasts antibodies. Multiple cycles of steroids and cyclosporine gave only transient responses and eventually she became transfusion dependent. DBA diagnosis was made after many years when exome sequencing became available. Similarly, in the second case, the patient was misdiagnosed with PRCA since childhood, and treated without benefit with intravenous immunoglobulin and steroids, with important side effects. Finally, exome sequencing found a classical RPS19 mutation consistent with DBA. At variance with the first two patients, diagnosis in our third patient was easier because he had an affected child and was a known carrier for a pathogenic DBA mutation. In this case, therapy with cyclosporine A led to a complete response.

Our cases mimic those reported in the literature, where diagnosis of congenital PRCA was made when genetic testing was not available. Nowadays, genetic for DBA should be always considered in adult patients with severe, transfusion-dependent, a-regenerative anemia without definitive cause. This recommendation is important as anemia might be a late manifestation in non-classical DBA and morphological abnormalities may be mild or completely absent, as observed in our cases. Importantly, not all cases are covered through current molecular testing and novel mutations of inherited BMFs are increasingly reported. However, the variable penetrance of the disease as well as the limited availability of molecular tests, underscores the importance of clinical suspicion. The latter remains crucial for orienting the diagnosis of DBA both in childhood and adulthood. Moreover, our third case, underlines the importance for active hematological follow up also in unaffected relatives of DBA patients. All in all, it is worth considering DBA among the differential diagnoses of unexplained isolated hyporegenerative anemia, even in adults and in the absence of syndromic features. Regarding bone marrow evaluation, the latter patient presented with well represented erythroid lineage, underlining the difficulty in the assessment of bone marrow histopathology in DBA.

Interestingly, all of our cases presented a moderate polyclonal T cell infiltrate, which accounted for the misdiagnosis of the more common PRCA. This poses the question of how many adult patients diagnosed with PRCA harbor a DBA mutation, without the classical phenotype. Consistently, two of our three patients responded to treatment with cyclosporine, which is the standard treatment for PRCA [15]. Conversely, treatment of DBA is usually based on steroids, while cyclosporine is not considered. Our results using the latter are slightly better than those reported in the literature, although the small number do not allow any definite comparison. However, the presence of a significant T-cell population in the bone marrow of all our three cases, may suggest the existence of an autoimmune *noxa* that contributes to the clinical picture and explains the potential effectiveness of cyclosporine A. Therefore, the latter may be taken into consideration in DBA patients not responding to steroids, particularly if presenting with a bone marrow T-cell lymphoid infiltrate. In addition, the single responding patient reported in the literature received concomitant treatment with danazol, an old therapy widely used in several autoimmune conditions and bone marrow failures, which recently received new consideration [16]. Regarding new treatments, the thrombopoietin receptor agonist eltrombopag induced a sustained hemoglobin increase and > 50% reduction of transfusion requirements in one out of 15 adult patients with DBA. This effect may be due to the activity of eltrombopag on the progenitor stem cell as well as on intracellular iron chelation. However, > 40% of patients required dose reductions due to asymptomatic thrombocytosis [17]. Another interesting agent may be the activin inhibitor luspatercept, which is able to restore late-stage erythropoiesis and to induce hematologic improvement in anemic patients with ineffective erythropoiesis (thalassemia and MDS). Importantly, chronic transfusion need in DBA patients may result in iron overload. Monitoring iron laboratory markers and cardiac and hepatic T2* MRI to guide stringent iron chelation therapy is of pivotal importance in these patients.

Finally, although no patient presented with secondary malignancy during the follow up, the increased risk for second solid and hematological cancers underlies the need for a long term surveillance of DBA patients, independently from the clinical response.

In conclusion, the availability of genetic testing led to an increased number of adult DBA diagnoses including those with non-canonical phenotype and silent carriers. The shaded borders between DBA and other bone marrow failures (particularly PRCA), require larger genetic testing in otherwise undiagnosed a/hypo-regenerative anemia and increased clinical awareness among adult hematologists.

## Data Availability

The data that support the findings of this study are not openly available due to reasons of sensitivity and are available from the corresponding author upon reasonable request. Data are located in controlled access data storage at Fondazione IRCCS Ca’ Granda Ospedale Maggiore Policlinico.
